# Preparation of a Cross-Linked Cartilage Acellular-Matrix Film and Its In Vivo Evaluation as an Antiadhesive Barrier

**DOI:** 10.3390/polym11020247

**Published:** 2019-02-02

**Authors:** Joon Yeong Park, Bo Ram Song, Jin Woo Lee, Seung Hun Park, Tae Woong Kang, Hee-Woong Yun, Sang-Hyug Park, Byoung Hyun Min, Moon Suk Kim

**Affiliations:** 1 Department of Molecular Science and Technology, Ajou University, Suwon 16499, Korea; pjy16@ajou.ac.kr (J.Y.P.); br8551@naver.com (B.R.S.); dlwlswlsdndn@ajou.ac.kr (J.W.L.); hpt88@ajou.ac.kr (S.H.P.); taipoong@ajou.ac.kr (T.W.K.); yuni_06@naver.com (H.-W.Y.); 2 Cell Therapy Center, Ajou University Medical Center, Suwon 16499, Korea; 3 Department of Biomedical Engineering, Pukyong National University, Busan 48513, Korea; shpark1@pknu.ac.kr

**Keywords:** antiadhesive film, cartilage acellular matrix, cross-linking, tissue adhesion

## Abstract

In this paper, a cartilage acellular-matrix (CAM) is chosen as a biomaterial for an effective antiadhesive barrier to apply between injured tissue and healthy tissues or organs. CAM is cross-linked using glutaraldehyde to create a cross-linked CAM (Cx-CAM) film. Cx-CAM has higher elastic modulus and toughness and more hydrophobic surface properties than CAM before cross-linking. Small intestinal submucosa (SIS), cross-linked SIS (Cx-SIS) as a negative control, and Seprafilm as a positive control are used in an experiment as adhesion barriers. Human umbilical vein endothelial cells (HUVECs) on SIS, Cx-SIS, or in a culture plate get attached and effectively proliferate for 7 days, but Cx-CAM and Seprafilm allow for little or no attachment and proliferation of HUVECs, thus manifesting antiadhesive and antiproliferative effects. In animals with surgical damage to the peritoneal wall and cecum, Cx-CAM and Seprafilm afford little adhesion and negligible inflammation after seven days, as confirmed by hematoxylin and eosin staining and macrophage staining, in contrast to an untreated-injury model, SIS, or Cx-SIS film. Cx-CAM significantly suppresses the formation of blood vessels between the peritoneal wall and cecum, as confirmed by CD31 staining. Overall, the newly designed Cx-CAM film works well as an antiadhesion barrier and has better anti-tissue adhesion efficiency.

## 1. Introduction

Injury of human tissue induces several physiological responses for self-healing of the injured tissue [1]. The physiological responses are a complex process: Infiltration by macrophages, increased permeability of blood vessels, fibrous tissue growth, and tissue repair [2]. This process can induce adhesion between the tissue (at the injury site) and the surrounding healthy tissues or organs through the formation of blood vessels and/or fibrinous adhesions.

Postoperative adhesions are quite common and are often a result of injury during and/or after the surgical procedure [3]. Adhesions can lead to serious complications such as a repeat surgical operation, postsurgical morbidity, chronic pain at the operated site, and a reduction in the quality of life of patients [4]. Although some surgical technologies are intended to minimize the formation of adhesions, postoperative adhesions seem to be an almost unavoidable consequence of surgical intervention.

Nevertheless, adhesions can be reduced by means of an antiadhesive barrier, which is a medical implant serving to separate the tissue (at the injury site) and healthy tissues or organs at the end of a surgical procedure [5,6,7]. To date, several antiadhesive barriers involving biomaterials such as sodium hyaluronate (HA), gelatin, carboxymethyl cellulose (CMC), extracellular matrices (ECMs), and polyester have been developed as a physical film, electrospun nanofiber membrane or a type of hydrogel and used as a commercial product to meet diverse clinical needs [8,9,10,11,12]. 

Recently, it was found that a cartilage acellular-matrix (CAM) can be easily prepared, it mainly consists of collagen and glycosaminoglycans, and it was tested as an ECM candidate for various biomedical applications [13,14,15,16]. Some, including our group, have reported that CAM inhibits neovascularization [17,18,19,20]. Because of this feature, it is conceivable that CAM will inhibit the formation of blood vessels at an injury site due to the antiadhesive properties of the proteoglycan-rich matrix. 

Physical film structures have been studied increasingly, and, as a result, antiadhesive films have been developed. A CAM powder can easily form a physical film, but a CAM film readily dissolves in biological media. Consequently, cross-linking of a CAM film needs to generate a stable antiadhesive barrier without the deformation under physiological conditions. Therefore, it was expected that a CAM film would be generated by a cross-linking reaction of randomly oriented and fibrillar CAM with a cross-linking agent. 

In this work, we prepared a cross-linked CAM (Cx-CAM) film using glutaraldehyde (GA) as a cross-linking agent to examine potential usability of the Cx-CAM film as an antiadhesive barrier because fabrication of the Cx-CAM film was expected to be the simplest and most cost-effective method. Furthermore, it is conceivable that the Cx-CAM film may serve as an effective antiadhesive barrier applied between an affected tissue (at an injury site) and healthy tissues or organs. 

Meanwhile, a small intestinal submucosa (SIS) derived from a porcine intestinal submucosal layer has already gained U.S. Food and Drug Administration (FDA) approval as a biomaterial for various biomedical applications [21]. SIS has a surface suitable for cell attachment and proliferation, as well as for the formation of blood vessels. Thus, we chose SIS and a film of SIS cross-linked by GA (Cx-SIS) as a negative control. As a positive control, Seprafilm (approved by FDA and comprised of two anionic polysaccharides of HA and CMC) was employed as an adhesion barrier in this work.

Usually, adhesions start to form within hours after a surgical procedure and may cause attachment between the affected tissue (at the injury site) and surrounding healthy tissues or organs through the formation of blood vessels and/or fibrinous adhesions within 3–7 days. Therefore, in this study, we examined the antiadhesive effect of the proposed material in an injured tissue at 7 days after an operation.

Accordingly, we investigated in vivo suitability of the Cx-CAM film as an antiadhesive material to elucidate the antiadhesive effect. Therefore, the present study had the following objectives: (1) To test whether the prepared Cx-CAM film has suitable mechanical and wetting properties as an antiadhesive film, (2) to determine whether the prepared Cx-CAM film has antiadhesive and antiproliferative effects against human umbilical vein endothelial cells (HUVECs), and (3) to see if the prepared Cx-CAM film has an antiadhesive effect in terms of inhibition of adhesions, suppression of macrophage infiltration, and downregulation of blood vessel formation in a rat model of a surgically damaged peritoneal wall and cecum.

## 2. Materials and Methods

### 2.1. Preparation of a CAM Powder

CAM tissues were harvested from market pigs (Finish pig, F1; Landrace + Yorkshire, ~100 kg at 6 months of age) within 6 h after euthanasia. The primitive knee joint tissues attached to the fore and hind legs of the obtained pigs were clipped off with a blade, and then placed in 1× phosphate-buffered saline (PBS) for 10 min. The obtained cartilaginous tissues were washed three times with deionized water (DW) and then frozen at −70 °C and 5 mTorr, followed by freeze-drying for 48 h. The fully dried primitive cartilaginous tissues were pulverized in a freezer mill (6700, SPEX Inc., Metuchen, NJ, USA) at −198 °C to obtain a CAM powder. For decellularization, the resultant CAM powder was mixed and stirred with a hypotonic buffer (10 mM Tris-HCl, pH 8.0) for 4 h at 4 °C and then stirred for 2 h at 4 °C in 1% sodium dodecyl sulfate in Tris-buffered saline (10 mM NaCl, pH 7.6).

A 50 mL aliquot of the resultant CAM suspension was placed in a 50 mL tube and centrifuged for 5 min at 2000 rpm, followed by removal of the supernatant. The obtained CAM tissues were mixed with DW (50 mL) and incubated with DNase (100 U μm^−1^, Elpis Biotech, Daejeon, Korea) for 12 h at 37 °C. After centrifugation for 10 min at 2000 rpm, the resultant CAM tissues were washed with DW and then were frozen at −70 °C and 5 mTorr and freeze-dried for 48 h to obtain a CAM powder with a particle size of 10–20 µm. The resulting CAM powder (1 g) was added into 20 mL vials containing an aqueous solution consisting of HCl (0.5 M) and pepsin (800 U mL^−1^) and then stirred for 24 h, followed by neutralization (pH 7.4) with a 4N NaOH solution. The resultant CAM suspensions were dialyzed for 24 h against DW, which was replaced at 1 h intervals. After that, the CAM suspensions were frozen at −70 °C and 5 mTorr and freeze-dried for 48 h to obtain a CAM powder. The dried CAM powder was finally pulverized in a freezer mill at −198 °C.

### 2.2. Preparation of a CAM Film (Before Cross-Linking) and Cx-CAM Film (After Cross-Linking)

The CAM powder (650 mg) was resuspended in DW (50 mL) to prepare a 1.3 wt % suspension, which was stirred for 48 h at room temperature. The CAM suspension was placed in a lab-made Teflon mold (50 × 50 mm, thickness 3 mm) and incubated at 37 °C for 24 h to obtain a CAM film.

To prepare Cx-CAM via GA (Sigma Chemicals, St. Louis, MO, USA), to the CAM film (6 mg) in a Teflon mold we added a 0.1% GA solution and then stirred it at 100 rpm for 1 h at room temperature. The remaining GA was removed from the Teflon mold using a pipette. Then, the Cx-CAM film was washed with PBS three times, incubated in DW, and stirred at 100 rpm for 30 min. Sequentially, a 4 m NaCl solution was added to the Cx-CAM film, followed by stirring at 100 rpm at room temperature. After 1 h, the remaining NaCl solution was removed from the Cx-CAM film. Next, the Cx-CAM film was washed four times with PBS and then four times with DW. GA concentration after washing was detected by high-performance liquid chromatography (HPLC). GA concentration after washing dropped below the detection limits in HPLC. The washed Cx-CAM film in the Teflon mold was dried at 37 °C for 24 h. After that, each Cx-CAM film was lyophilized until the residue reached a constant weight in a freeze dryer. The obtained Cx-CAM maintained almost the same weight with initial CAM (not exceeding 2% difference). The swelling ratios of obtained Cx-CAM film and Seprafilm determined and added in the Supplementary Information. CAM and Cx-CAM films were cut into discs with a diameter of 6 mm and thickness 50 µm. 

### 2.3. Preparation of a SIS Film (Before Cross-Linking) and Cx-SIS Film (After Cross-Linking)

SIS film was prepared according to previously reported methods [21]. The SIS cross-linked by means of GA (Cx-SIS) was prepared by the same procedure as described in Section 2.2. 

### 2.4. Incubation of CAM, Cx-CAM, SIS, and Cx-SIS Films in PBS

Each film was separately immersed in PBS (8 mL) in a 20 mL vial and incubated at 37 °C with shaking at 100 rpm for 7 days (*n* = 3 for each data point). The shape of each film was visualized with a Handheld digital microscope (AM3713TB Dino-Lite Premier, Dino-Lite, New Taipei City, Taiwan).

### 2.5. Mechanical Properties of CAM, Cx-CAM, SIS, and Cx-SIS Films

All films were cut into rectangular pieces with dimensions 15 mm × 30 mm × 50 µm. The stress–strain and tensile strengths of all the films were measured on a Universal testing machine (H5KT, Tinius-Olsen, Horsham, PA, USA) at a feed rate of 1 mm min^−1^ with a force of 50 N. Elastic modulus of each film was calculated from the slope of the linear region (0.1%–4% strain) of the stress–strain curve of each film. The toughness of each film was calculated analytically from the area under the stress–strain curve. Three samples in each film group were analyzed separately. The results were presented as mean ± standard deviation (SD).

### 2.6. Contact Angles of CAM, Cx-CAM, SIS, and Cx-SIS Films

Water contact angles of all the films and Seprafilm were measured by the sessile drop method at room temperature with an optical bench-type contact angle goniometer (Phoenix 150, Suwon, Korea). One droplet of purified water (10 µL) was deposited onto each film surface via a microsyringe attached to the goniometer. The water contact angle was measured within 5 s. Contact angle images were captured by a CCTV camera (XC-75, SONY, Tokyo, Japan) and then were measured in the ImageJ software (National Institutes of Health, Bethesda, MD, USA). Three samples in each film group were analyzed separately. The results were presented as mean ± SD.

### 2.7. Viability of HUVECs Cultured on a CAM, Cx-CAM, SIS, or Cx-SIS Film

To examine the adhesion-inhibitory effects of CAM, Cx-CAM, SIS, and Cx-SIS films on HUVECs, these cells (InnoPharmaScreen, Asan, Korea) were cultured in a 75 cm^2^ tissue culture flask (BD Falcon; San Jose, CA, USA) in a HUVEC growth medium (EGM-2 bulletkit cc-3156 & cc-4176, Lonza, Basel, Switzerland) at 5% CO_2_ and 37 °C.

The cultured HUVECs (10^5^ cells per well) were individually seeded on each CAM, Cx-CAM, SIS, or Cx-SIS film in the form of a disk of 6 mm diameter and on Seprafilm (Genzyme Biosurgery; Framingham, MA, USA), all placed in wells of a 24-well tissue culture plate (Falcon, Pittsburgh, PA, USA) and incubated for 4 h. One milliliter of the HUVEC medium was added into the wells of the 24-well tissue culture plates and changed every 3 days, and the cells were incubated for 1, 4, and 7 days. 

A 3-(4,5-dimethylthiazol-2-yl)-2,5-diphenyl tetrazolium bromide (MTT) assay was conducted to evaluate the viability of HUVECs on each film and on Seprafilm. After 1, 4, and 7 days, 100 μL of MTT (Sigma, St. Louis, MO, USA) in PBS was added to each film in each well. After 4 h at 37 °C, the added solution was removed from each well. After that, 500 μL of DMSO was added into each well and incubated for 30 min. One hundred microliters of the formed formazan solution were transferred into 96-well plates (SPL Lifescience, Gyeonggi-do, Korea). Finally, absorbance was measured on a plate reader (EL808 Ultra microplate reader, Bio Tek Instruments, Winooski, VT, USA) at a wavelength of 570 nm. Assays of the viability of HUVECs on each film were conducted three times.

### 2.8. Fluorescent Imaging of HUVECs Cultured on a CAM, Cx-CAM, SIS, or Cx-SIS Film

To monitor adhesion of HUVECs by fluorescent imaging, HUVECs were labeled by means of the PKH67 Green Fluorescent Cell Linker Kit (Sigma) as follows. Cultured HUVECs were washed three times with a serum-free medium and centrifuged at 2000 rpm for 5 min. HUVECs (10^7^) were employed to prepare a cell suspension by adding 500 μL of diluent C, mixed with the PKH67 stock solution (4.0 × 10^−6^ m) in diluent C (500 μL) and were incubated at 25 °C for 5 min. One milliliter of fetal bovine serum (Gibco, Grand Island, NY, USA) was added, and the solution was incubated at 25 °C for 1 min to stop the labeling reaction. Two milliliters of the complete HUVEC medium was added to dilute the latter cell suspension. The supernatant was discarded, and the cell pellet was washed two more times with the complete HUVEC medium (10 mL) to ensure removal of the unbound dye. The PKH67-labeled HUVECs were centrifuged at 2000 rpm for 10 min and washed three times with the HUVEC medium.

To examine fluorescent imaging of HUVECs on each type of film (CAM, Cx-CAM, SIS, or Cx-SIS, or Seprafilm), PKH67-labeled HUVECs (10^5^ cells per well) were individually seeded on a disk of film (6 mm diameter) placed in a 24-well tissue culture plate and incubated for 4 h. One milliliter of the HUVEC medium was added into the wells of the 24-well tissue culture plates and changed every 3 days, and PKH67-labeled HUVECs were incubated for 1, 4, and 7 days. PKH67-labeled HUVECs on all films were visualized in a dark environment under an inverted fluorescent microscope (Eclipse TS100, Nikon, Tokyo, Japan). 

### 2.9. Morphology of HUVECs Cultured on a CAM, Cx-CAM, SIS, or Cx-SIS Film

For scanning electron microscopy (SEM) analysis, HUVECs (10^5^ cells per well) were cultured in a 24-well plate. Each film and Seprafilm was taken out after 1, 4, and 7 days. HUVECs were fixed with 4% paraformaldehyde for 1 h and dehydrated in a graded series of ethanol solutions (70%, 80%, 90%, and 100%) for 10 min each. Next, the films were freeze-dried for 3 days and coated with a thin layer of gold using a plasma-sputtering apparatus (Emitech, K575, Kent, UK). Finally, the samples were analyzed under a JSM-6380 scanning electron microscope (JEOL, Tokyo, Japan).

### 2.10. Implantation into Animals

The protocols of this study were approved by the Institutional Animal Experiment Committee (approval No. 2016-0011) at Ajou University School of Medicine. Eight-week-old Sprague–Dawley rats (weight: 320–350 g, males) were used in this study. 

The Sprague–Dawley rats were anesthetized by intramuscular injection with a 1:1 mixture of tiletamine and zolazepam (Zoletil^TM^ 50; Virbac, Carros, France) and xylazine (Rompun; Bayer Korea, Seoul, Korea; 1 mL kg^−1^). A 4 cm L-shape incision was made along the abdominal midline, and a bleeding lesion of 1.0 × 1.0 cm was created on the peritoneal and cecal surface by means of Rochester-Pean forceps. In the experimental group, the two injured surfaces were covered with a 1.5 × 1.5 cm CAM, Cx-CAM, SIS, Cx-SIS, or Seprafilm. After the wound area was completely covered, four corners were sutured with braided absorbable suture 6–0 (0.7 metric) (Covidien, Dublin, Ireland) between the peritoneum and the cecum to prevent movement of implanted films. The control group consisted of Sprague–Dawley rats with untreated defects. The incisions of all animals were closed in muscle and skin layers with monofilament absorbable suture 3–0 (2 metric; Covidien, Dublin, Ireland), and all rats were maintained on normal food and water for 7 days. After that, all Sprague–Dawley rats were euthanized under anesthesia. After laparotomy was performed, the suture at four corners between the damaged peritoneum and the cecum was removed by straight surgical scissors to evaluate the anti-tissue adhesive effect. The antiadhesion effect of the films was evaluated by three observers in a blinded manner according to the classification of Mazuji et al. [22] The following antiadhesion scoring system was implemented: grade 0 = no adhesion; grade 1 = very thin, easily separable intestinal adhesion; grade 2 = moderately dense, scattered adhesion; grade 3 = dense, continuous adhesion; and grade 4 = very dense homogeneous adhesion. All antiadhesion scores were evaluated in a blinded manner by three raters at the same time points, and the results are represented as mean ± SD.

### 2.11. Histological Analysis

Seven days after animal transplantation, samples of intestinal and muscle layers were fixed in 10% formaldehyde for 48 h at room temperature. The fixed samples were embedded in paraffin and cut into 5-micrometer-thick slices. The tissue section slides were incubated in a 60 °C oven for 2 h to remove paraffin. For hematoxylin and eosin (HE) staining, the slides were deparaffinized in xylene for 10 min to remove remaining paraffin and hydrated in a graded series of ethanol solutions (100%, 95%, 90%, 80%, and 70%) for 10 min each. The hydrated slides were stained with a hematoxylin solution (Sigma) for 5 min and rinsed with DW for 2 min. Then, they were stained with a 20% eosin solution (Sigma) for 5 min and rinsed with DW for 1 min. The stained slides were dehydrated with a graded series of ethanol (70%, 80%, 90%, and 100%) for 30 sec each time. After that, the slides were fixed and mounted with a mounting medium (Muto Pure Chemicals, Tokyo, Japan).

For immunohistochemical staining (for ED1), the slides were deparaffinized in xylene for 10 min to remove the remaining paraffin and hydrated in a graded series of alcohol (100%, 95%, 90%, 80%, and 70%) for 10 min each time. The hydrated slides were incubated in citrate buffer (Sigma) at 120–130 °C for 10 min. The incubated slides were washed twice with PBS for 5 min and with PBST (0.05% Tween 20 in PBS) for 10 min. The slides were blocked with 5% horse serum (Gibco, Auckland, New Zealand) and 5% bovine serum albumin (BSA; Bovogen, Victoria, Australia) in PBS for 90 min at 37 °C. After that, the blocked slides were washed twice with PBS for 5 min and PBST for 10 min. The slides were incubated with antibody ED1 (mouse anti-rat CD68; Serotec, Oxford, UK) in an antibody diluent (DAKO, Glostrup, Denmark; 1:1000) for 16 h at 4 °C and then washed twice with PBS for 5 min and PBST for 10 min. The washed slides were blocked with 1% BSA in PBS for 30 min and then incubated with a secondary antibody (a goat anti-mouse IgG antibody conjugated with Alexa Fluor 594; Invitrogen, Carlsbad, CA, USA) in 1% BSA (1:200) for 3 h at 25 °C. The incubated slides were washed twice with PBS for 5 min and PBST for 10 min. Afterward, the slides were mounted via the Pro-Long Gold Antifade Reagent with DAPI (Life Technologies, Grand Island, NY, USA).

For CD31 staining, the deparaffinized slides were hydrated in the same way as in the HE and ED1 staining above. The hydrated slides were incubated in citrate buffer at 120–130 °C for 10 min. The incubated slides were washed twice with PBS for 5 min and PBST for 10 min. The slides were blocked with 5% horse serum and 5% BSA in PBS for 90 min at 37 °C. After that, the blocked slides were washed twice with PBS for 5 min and PBST for 10 min. The slides were incubated with an anti-CD31 antibody (mouse anti-rat CD31; Abcam, Cambridge, UK) in antibody diluent (DAKO, Glostrup, Denmark; 1:200) for 16 h at 4 °C and then washed twice with PBS for 5 min and PBST for 10 min. The washed slides were blocked with 1% BSA in PBS for 30 min and then incubated with a secondary antibody (a goat anti-mouse IgG antibody conjugated with Alexa Fluor 594; Invitrogen) in 1% BSA (1:200) for 3 h at 25 °C. The incubated slides were washed twice with PBS for 5 min and PBST for 10 min. Next, the slides were mounted via the Pro-Long Gold Antifade Reagent with DAPI (Life Technologies). The percentage of CD31-positive stained images was determined by dividing the mean number of CD31-positive stained images by the mean number of DAPI positive cells.

All the stained slides were visualized on Axio Imager A1 (Carl Zeiss Microimaging GmbH, Göttingen, Germany) equipped with Axiovision software (Rel. 4.8; Carl Zeiss Microimaging GmbH).

### 2.12. Statistical Analysis

All data (*n* = 3) on tensile strength, contact angle measurements, MTT assays, adhesion scoring, adhesion thickness, ED1 assay, and CD31 assay are presented as the mean ± SD. The data were subjected to one-way analysis of variance (ANOVA) with Bonferroni’s post hoc test in SPSS 12.0 software (SPSS Inc., Chicago, IL, USA).

## 3. Results and Discussion

### 3.1. Preparation of CAM Powders

Primitive cartilage tissue as a raw material was first collected from the porcine knee cartilage of fore and hind legs. The mechanical manipulation and decellularization of cartilage tissue were carried out to remove cartilage-like tissue components and DNA (derived from cartilage cells and blood cells). 

**Figure 1:**
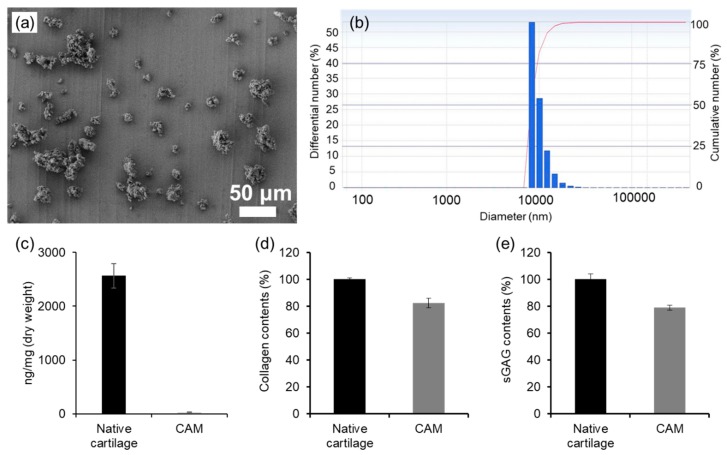
(**a**) SEM mages and (**b**) particle size of cartilage acellular-matrix (CAM) powder, and (**c**) DNA, (**d**) collagen, and (**e**) glycosaminoglycans contents of native cartilage and CAM.

After processing, the obtained CAM powders had a particle size of 10.5 ± 2.5 μm (Figure 1a,b). Additionally, DNA was not detectable, implying perfect removal of all cartilage cells (Figure 1c). Furthermore, the obtained CAM powders almost retained the collagen and glycosaminoglycans contents of original cartilage matrix (Figure 1d,e). The obtained CAM powders were not soluble in physiological saline. Thus, after treatment with a mixture of HCl and pepsin aqueous solutions, the CAM powder was solubilized in a suspension form. Overall, the CAM suspension was prepared successfully as a biomaterial for preparation of a CAM antiadhesive film.

### 3.2. Preparation of CAM and Cx-CAM Antiadhesive Films

The obtained CAM suspension was poured into the molds and dried at 37 °C to form the CAM film. Next, to prepare a Cx-CAM film, the CAM film was cross-linked by means of GA because GA can react with the amine groups between and within CAM chains. After cross-linking, the Cx-CAM film was washed with PBS to remove unreacted GA. No GA peak was detected by HPLC after washing of the Cx-CAM film (GA concentration dropped below the detection limits in HPLC: Supplementary Information Figure S1). 

**Figure 2:**
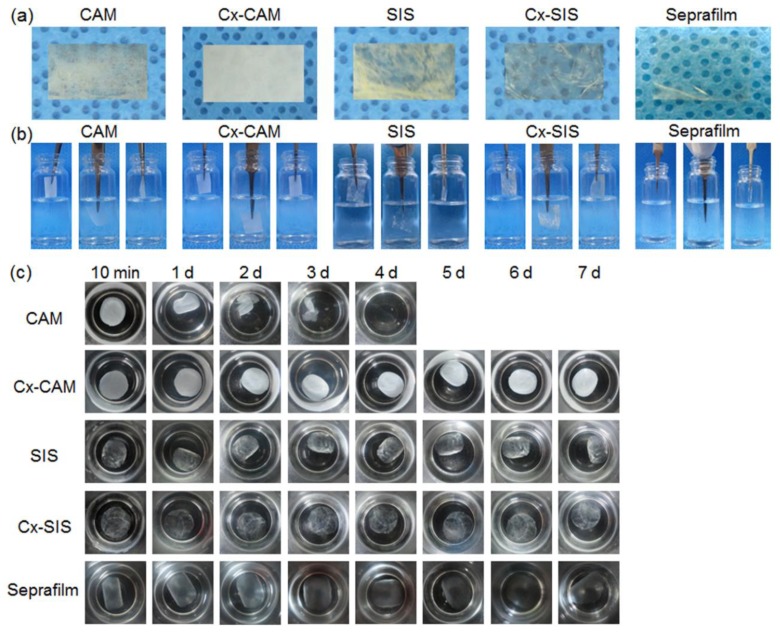
(**a**) Images of (**b**) side and (**c**) top views of CAM, cross-linked CAM (Cx-CAM), small intestinal submucosa (SIS), cross-linked SIS (Cx-SIS) films and Seprafilm before and after incubation in a vial with PBS.

The obtained film had an almost uniform thickness of 50 µm in adjustable parameters. The resultant CAM and Cx-CAM films were translucent as presented in Figure 2. In SEM, CAM exhibited agglomerated or connected fiber of native CAM powder, but Cx-CAM showed flat film shape with roughness (Supplementary Information Figure S2).

In PBS, the CAM film did not maintain the film shape because it started to dissolve after 1 h and then got completely solubilized within 2–3 days. In contrast, the Cx-CAM film maintained the film shape in PBS, and was not soluble in this PBS because the amine groups within and between CAM chains were cross-linked by GA. Meanwhile, SIS and Cx-SIS films swelled in water as well as in physiological saline and were not soluble. The reason was probably that the native triple-helical SIS fibril structures did not dissolve. 

Collectively, these results indicate that the Cx-CAM was successfully prepared as an antiadhesive-barrier for future cellular and animal experiments. 

### 3.3. Properties of CAM, Cx-CAM, SIS, and Cx-SIS Antiadhesive Films

**Figure 3:**
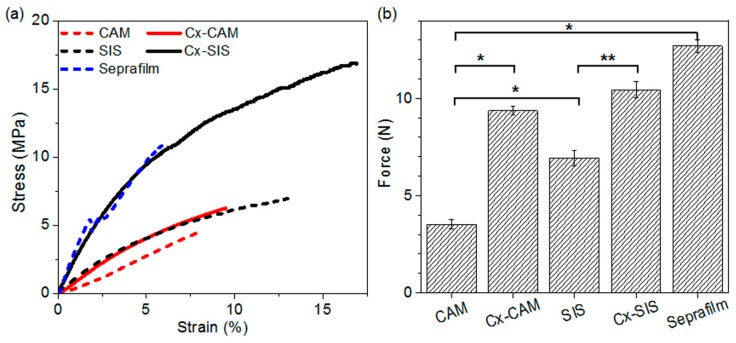
(**a**) Stress–strain curves and (**b**) tensile strengths of CAM, Cx-CAM, SIS, Cx-SIS films, and Seprafilm. (* *p* < 0.001, ** *p* < 0.05).

The mechanical properties of CAM, Cx-CAM, SIS, and Cx-SIS antiadhesive films and Seprafilm were determined using a universal testing machine (Figure 3). Elastic modulus and toughness were calculated from the strain–stress curves of CAM, Cx-CAM, SIS, and Cx-SIS films. 

In a strain–stress curve, the linear portion is the elastic region, and thus the slope indicates the elastic modulus of CAM and SIS films (Supplementary Information Figure S3). The elastic moduli of these films before cross-linking were 59.2 ± 0.1 and 81.6 ± 0.6 MPa, respectively. The elastic moduli of the Cx-CAM and Cx-SIS films were significantly higher, at 66.0 ± 0.5 and 240.2 ± 1.2 MPa, respectively. Because of cross-linking, Cx-CAM and Cx-SIS films showed 1.1- and 2.9-fold greater resistance to elastic deformation judging by applied stress. The elastic moduli of Seprafilm were 327.3 ± 1.0 MPa. Toughness refers to how much energy each film can absorb before rupturing. Toughness of Cx-CAM and Cx-SIS films (0.35 and 1.92 MPa) was higher than that of CAM and SIS (0.19 and 0.61 MPa, respectively). Toughness of Seprafilm was 0.4 MPa. The CAM and SIS films before cross-linking had a tensile strength of 3.5 and 6.9 N, respectively. After cross-linking, Cx-SIS had higher tensile strength, 10.4 N. The tensile strength of Cx-CAM was also significantly greater: 9.4 N. 

Although these results showed different mechanical properties between Cx-CAM and Seprafilm owing to the different fabrication using different biomaterials, collectively we found that the cross-linking procedure can fortify the mechanical properties of CAM. 

**Figure 4:**
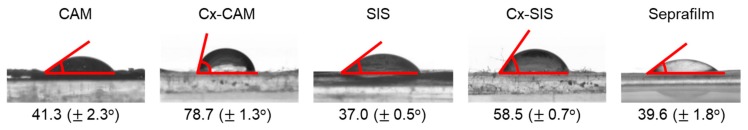
Images of a static droplet (side view) and contact angles of CAM, Cx-CAM, SIS, Cx-SIS films, and Seprafilm.

Next, the contact angles of CAM, Cx-CAM, SIS, and Cx-SIS antiadhesive films were measured to compare surface wetting properties (Figure 4). Seprafilm showed contact angles of 39.6°. CAM and SIS had contact angles of 41.3° and 37° before cross-linking. The contact angles of Cx-CAM and Cx-SIS were greater, at 78.7° and 58.5°. The cross-linking reaction resulted in an increase in contact angles. The contact angle change was probably due to the morphology difference of CAM and Cx-CAM (Supplementary Information Figure S2). This result is indicative of the properties of an antiadhesive film that is more hydrophobic because of the cross-linking reaction. Additionally, Cx-CAM had more hydrophobic surface properties than Cx-SIS, although hydrophobic Cx-CAM can induce adsorption of albumin and uncontrolled interactions. 

Next, the swelling ratios of Cx-CAM and Seprafilm were compared (Supplementary Information Figure S4). Seprafilm absorbed 860% of PBS at 1 h, 1470% at 6 h, and reached 1640% at 12 h, indicating the absorption of a large amount of a biological medium. Meanwhile, Cx-CAM absorbed 280% of PBS at 1 h, 330% at 3 h, and reached and maintained 400% at 6–12 h. This result indicated that Cx-CAM can absorb a suitable amount of a biological medium.

Thus, we confirmed that mechanical and wetting properties of CAM-based antiadhesive films were affected by the cross-linking reaction. 

### 3.4. Antiadhesive and AntiproliferAtive Effects on HUVECs Seeded on Antiadhesive Films

Because HUVECs are popular as an experimental model of vessel formation, the viability rates of HUVECs on CAM, Cx-CAM, SIS, and Cx-SIS films were examined for 7 days by the MTT assay. Seprafilm and a culture plate served as controls. 

**Figure 5:**
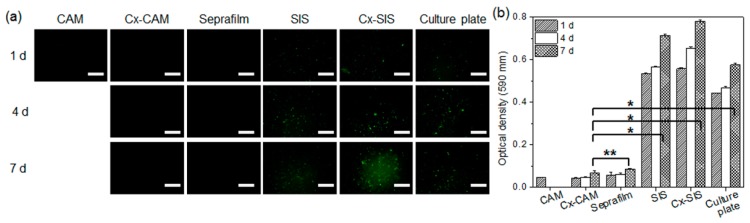
(**a**) PKH67 green fluorescence images and (**b**) MTT assays of HUVECs cultured on CAM, Cx-CAM, SIS, Cx-SIS, Seprafilm, or a culture plate for 1, 4, or 7 days (magnification is 200×, and the scale bar represents 100 μm; * *p* < 0.001, ** *p* > 0.99).

First, fluorescent images of PKH67-labeled HUVECs were examined to confirm the attachment and proliferation of HUVECs (Figure 5a). Green fluorescent dot images, assignable to PKH67-labeled HUVECs, were examined. 

There were few or no green fluorescent dots on CAM, Cx-CAM, and Seprafilm, implying the adhesion-inhibitory effects on HUVECs. PKH67-labeled HUVECs on CAM were quantified only at the time point “1 day” because CAM completely dissolved after 1 day as described in a Section 3.2. Meanwhile, the green fluorescent dots were seen on a culture plate, SIS, and Cx-SIS film after 1 day and increased with incubation time. 

A quantitative MTT assay was performed to evaluate the attachment and proliferation of HUVECs (Figure 5b). The culture plate, SIS, and Cx-SIS contained strongly attached HUVECs after 1 day and gradually proliferating HUVECs for 7 days. Meanwhile, MTT assays of cells on CAM, Cx-CAM, and Seprafilm revealed a significantly lower (*p* < 0.001) optical density compared with the culture plate, SIS, and Cx-SIS after 1, 3, and 7 days of incubation. MTT assays of cells on CAM showed very low optical density as determined at 1 day after initiation of the experiment because CAM completely dissolved after 1 day as described in a Section 3.2. 

This finding indicated that Cx-CAM yielded approximately 90% inhibition as compared to the culture plate group. Results of quantitative MTT assays of cells on the Cx-CAM and Seprafilm did not differ significantly (*p* > 0.99). This result indicates that Cx-CAM significantly inhibits the attachment and proliferation of HUVECs.

**Figure 6:**
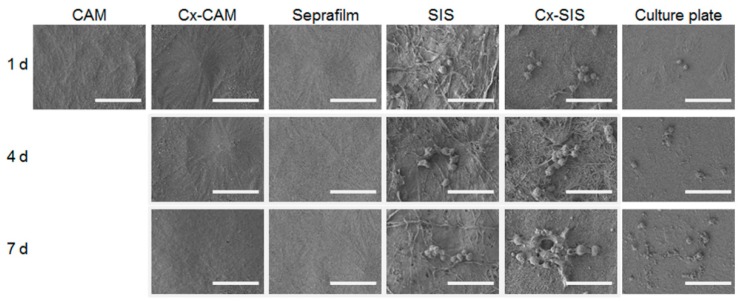
SEM images of HUVECs cultured on CAM, Cx-CAM, SIS, Cx-SIS, Seprafilm, or a culture plate for 1, 4, or 7 days (magnification is 1000×, and the scale bar represents 50 μm).

The HUVECs cultured on CAM, Cx-CAM, Seprafilm, SIS, Cx-SIS, or the culture plate were examined by SEM (Figure 6). HUVECs got attached to the culture plate, SIS, and Cx-SIS after 1 day, and effectively proliferated for 7 days, indicating little or no inhibition of the attachment and proliferation of HUVECs. In contrast, CAM, Cx-CAM, and Seprafilm allowed for little or no attachment and proliferation of HUVECs, meaning inhibition of the attachment and proliferation of HUVECs.

Collectively, these results indicated that Cx-CAM acted as an antiadhesive film that inhibits the attachment and proliferation of HUVECs. 

### 3.5. In Vivo Anti-Tissue Adhesion Effects of CAM, Cx-CAM, SIS, and Cx-SIS Antiadhesive Films

**Figure 7:**
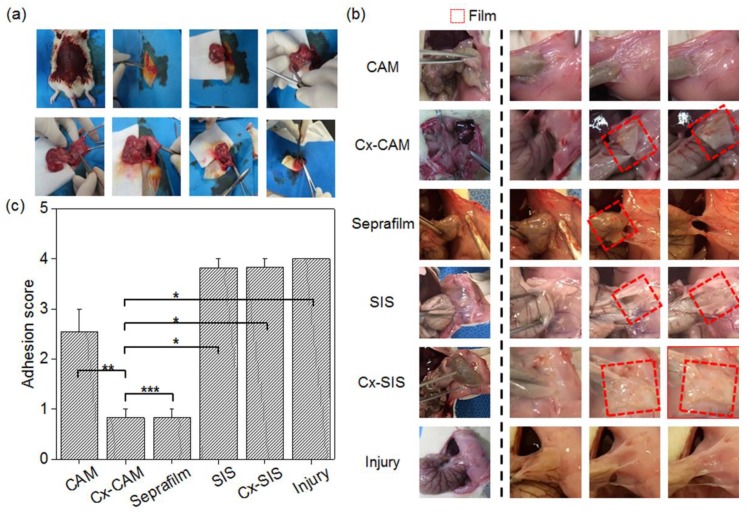
(**a**) The surgical procedure that was performed to set up the animal model, (**b**) images of CAM, Cx-CAM, SIS, Cx-SIS, Seprafilm, and nontreatment (injury) and (**c**) an in vivo scoring test of CAM, Cx-CAM, Seprafilm, SIS, Cx-SIS, and the injury model without a film 7 days after the implantation (* *p* < 0.001, ** *p* < 0.05, *** *p* > 0.99).

The rats were surgically damaged on the peritoneal wall and cecum to evaluate the in vivo anti-tissue adhesion influence of CAM, Cx-CAM, Seprafilm, SIS, or Cx-SIS. All antiadhesive films were easily and safely implanted between the surgically damaged peritoneal wall and cecum (Figure 7a).

After 7 days, the adhered tissue between the peritoneum and cecum was characterized by visual observation (Figure 7b). Animals without the antiadhesive film—as an injury model—showed completely adhered tissue between the peritoneum and cecum. The rats with an implanted SIS or Cx-SIS film also showed completely adhered tissue on the surfaces of the peritoneum and cecum. 

CAM implant rats showed slightly adhered tissue and no film between the peritoneum and cecum, owing to the quick degradation as described in a Section 3.2. By contrast, in Cx-CAM and Seprafilm implant groups, the peritoneum and cecum were easily detached, and smooth surfaces of the peritoneum and cecum were clearly seen. Each implanted Cx-CAM or Seprafilm film was detectable between the peritoneum and cecum. This result revealed that Cx-CAM or Seprafilm film served well as an outstanding antiadhesive film between the peritoneum and cecum for 7 days.

To quantitatively determine the degree of tissue adhesion, grading was performed by pulling the cecum from the clogged peritoneal wall (see the Experimental section; Figure 7c). Animals without an antiadhesive film as the injury model and with an implant of SIS or Cx-SIS scored 4.0, 3.8, and 3.8 points, respectively. The CAM implant group got an adhesion score of 2.5 points. This finding indicates that CAM acted as an antiadhesive film in the early period but did not retain the antiadhesive effect for 7 days because of the degradation. 

Cx-CAM or Seprafilm film got the same score of 0.8 points. This result revealed that—as an antiadhesive film—Cx-CAM or Seprafilm perfectly separated the damaged peritoneal wall and cecum for 7 days. 

Collectively, these results are consistent with the finding that CAM itself possesses a good antiadhesion effect, and Cx-CAM persisted for the tested experimental periods to effectively prevent tissue adhesion after the surgical procedure. 

### 3.6. Histological Analysis of In Vivo Implants

The adhesion thickness of adhered tissue and inflammation and blood vessels in the adhered tissue between the peritoneal wall and cecum of the rats that received an implant of CAM, Cx-CAM, Seprafilm, SIS, or Cx-SIS were evaluated by histological staining. 

**Figure 8:**
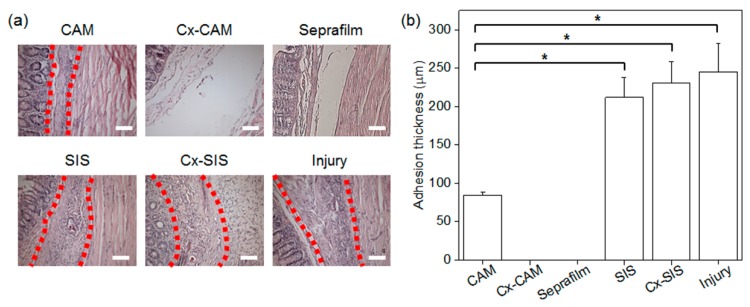
(**a**) HE staining (magnification is 200×, and the scale bar represents 100 μm) and (**b**) average adhesion thickness in experimental animals with an implant of CAM, Cx-CAM, Seprafilm, SIS, or Cx-SIS and in the injury model (without a film) after 7 days (* *p* < 0.01).

HE staining of all the implants clearly distinguished them from the peritoneal wall and cecum (Figure 8a). HE staining of the group without the antiadhesive film and of the groups with SIS or Cx-SIS film revealed extensive adhered tissue between the peritoneal wall and cecum. CAM also yielded adhered tissue but less. By contrast, HE staining of implants of Cx-CAM or Seprafilm detected no adhered tissue between the peritoneal wall and cecum.

Consequently, the thickness of the adhered tissue between the peritoneal wall and cecum was measured (Figure 8b). The group without an antiadhesive film and groups with SIS or Cx-SIS were found to have an adhesion thickness ranging between 210 and 245 µm. The adhesion thickness in the CAM implant group was smaller: 85 µm. There was zero adhesion thickness in groups with an implant of Cx-CAM or Seprafilm.

**Figure 9:**
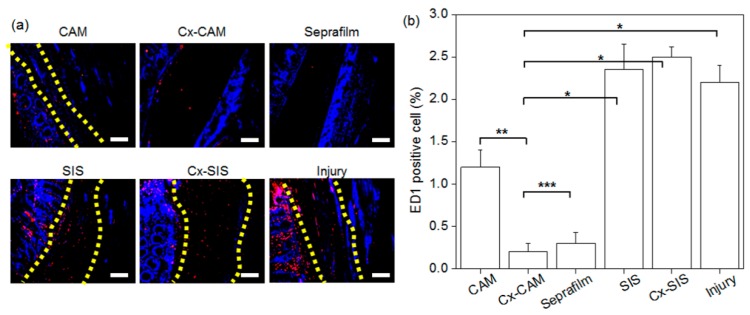
(**a**) ED1 immunofluorescent staining (magnification is 200×, and the scale bar represents 100 μm) and (**b**) the counts of ED1-positive cells in experimental animals with an implant of CAM, Cx-CAM, Seprafilm, SIS, or Cx-SIS and in the injury model (without a film) after 7 days (* *p* < 0.001, ** *p* < 0.05, *** *p* > 0.99).

Next, we performed macrophage ED1 staining to determine an inflammatory response of a host tissue between or on the peritoneal wall and cecum against the implanted antiadhesive films. This is because the macrophage-specific ED1 antibody staining is a unique in vivo indicator of the inflammatory response. Accordingly, all implants of CAM, Cx-CAM, Seprafilm, SIS, or Cx-SIS were next analyzed by immunohistochemical ED1 staining (Figure 9a). After ED1 immunofluorescent staining, red fluorescence was attributed to the macrophage marker CD68, and blue fluorescence to nuclei of live cells. The blue fluorescent signals were evident in all the implants. 

The group without an antiadhesive film and groups with SIS or Cx-SIS manifested abundant red fluorescence in adhered tissue between the peritoneal wall and cecum. For the implant of CAM, the red fluorescence was also present in the adhered tissue, but less in comparison with groups SIS and Cx-SIS. In contrast, hardly any red fluorescence was detectable in implants of Cx-CAM or Seprafilm, thereby indicating decreased numbers of macrophages. 

ED1-positive cells were counted, and the result was normalized to the total stained tissue area to determine the extent of macrophage infiltration (Figure 9b). The macrophage percentage in the injury model and in SIS and Cx-SIS implant models contained ED1-positive cells at 5.6%–6.1%. We conjectured that macrophages can be transitorily infiltrated inside adhered tissue between damaged peritoneal wall and cecum. However, this percentage in groups Cx-CAM and Seprafilm was lower: 0.6% and 0.7%, respectively. Cx-CAM yielded the smallest number of macrophages. This finding indicates that Cx-CAM and Seprafilm induced a lesser inflammatory response as compared with SIS and Cx-SIS. 

Finally, we evaluated the formation of blood vessels in the adhered tissue between or on the peritoneal wall and cecum because injured tissue can induce the formation of fibrous tissue bridges and blood vessels between tissues at the injury site and surrounding healthy tissues or organs as physiological responses. 

**Figure 10:**
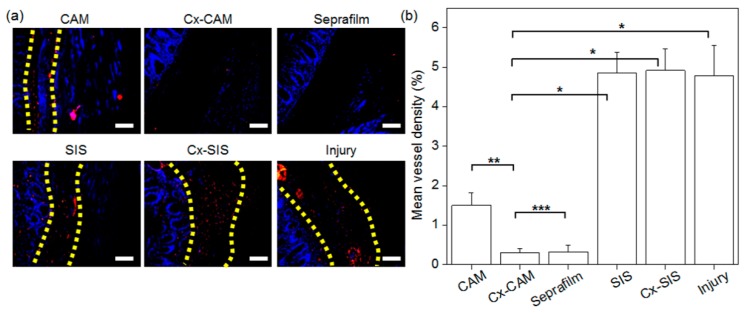
(**a**) CD31 staining (magnification is 200×, and the scale bar represents 100 μm) and (**b**) mean density of counted blood vessels in experimental animals with an implant of CAM, Cx-CAM, Seprafilm, SIS, or Cx-SIS and in the injury model (without a film) after 7 days (* *p* < 0.001, ** *p* < 0.05, *** *p* > 0.99).

All implants of CAM, Cx-CAM, Seprafilm, SIS, or Cx-SIS were stained with an anti-CD31 antibody (Figure 10a). The red staining was attributed to CD31-positive blood vessels. The group without an antiadhesive film and groups with SIS or Cx-SIS manifested abundant CD31-positive red fluorescence. CAM yielded a slightly lower intensity of red fluorescence in the adhered tissue. However, hardly any red fluorescence was detectable in the implants of Cx-CAM or Seprafilm, indicating inhibition of blood vessel formation. 

In the quantification of the amount of CD31-positive red staining (Figure 10b), the injury group without an antiadhesive film and groups with a SIS or Cx-SIS implant showed 5.4%, 5.6%, and 5.6% of CD31-positive red staining, respectively. The implants of CAM showed 2.9% (weaker) CD31 staining. Meanwhile, this percentage in groups Cx-CAM or Seprafilm was much lower, at almost the same value: 0.5% (Figure 10b). These results suggested that Cx-CAM or Seprafilm significantly suppressed the formation of blood vessels between the peritoneal wall and cecum, meaning the prevention of tissue adhesion. 

Overall, the Cx-CAM film possesses better anti–tissue adhesion efficiency for successful prevention of tissue adhesion after a surgical procedure.

## 4. Conclusions

In this work, we prepared a Cx-CAM antiadhesive film with greater elastic modulus and toughness and more hydrophobic surface properties in comparison with CAM before cross-linking. The Cx-CAM film has antiadhesive and antiproliferative effects on HUVECs. Moreover, Cx-CAM film successfully inhibits tissue adhesions, infiltration by macrophages, and the formation of blood vessels. Therefore, the Cx-CAM film as an antiadhesion barrier is well suited to additional preclinical studies involving a large-animal model although we should confirm that no free aldehyde group remained in Cx-CAM and establish the target mechanical property of Cx-CAM.

## Supplementary Materials

The following are available online at http://www.mdpi.com/2073-4360/11/2/247/s1.
