# Supplementary material for: Preparation of a Cross-Linked Cartilage Acellular-Matrix Film and Its In Vivo Evaluation as an Antiadhesive Barrier
Source: Polymers (Basel). 2019 Feb 2;11(2):247. doi: 10.3390/polym11020247 (PMC6419041; doi:10.3390/polym11020247)
Supplement: Supplementary file 1 [file polymers-11-00247-s001.pdf]

Supplementary Information

Preparation of a Cross-Linked Cartilage Acellular-Matrix Film and its in vivo Evaluation as an Antiadhesive Barrier

Joon Yeong Park, Bo Ram Song, Jin Woo Lee, Seung Hun Park, Tae Woong Kang, Hee-Woong Yun, Sang-Hyug Park, Byoung Hyun Min* and Moon Suk Kim*

Determination of the swelling property of Cx-CAM

The prepared Cx-CAM or Seprafilm (6 mg) were individually added into 80 mL vial, then we added 10 mL of PBS and incubated the mixtures at 37 °C. At predetermined time points, PBS that was not absorbed by Cx-CAM or Seprafilm was removed from the vials. The remaining PBS on the surface of vials was eliminated by clean wipers (Yuhan-Kimberly, Seoul, Korea). After that, the vial was weighed to measure the weight of wet Cx-CAM or Seprafilm. ~~After that,~~Then each Cx-CAM or Seprafilm was lyophilized until the residue reached a constant weight in a freeze dryer. The vial was weighed to determine the weight of dry Cx-CAM or Seprafilm.

The swelling ratio was defined as follows: Swelling ratio (%) = [(weight of wet Cx-CAM or Seprafilm – weight of dry Cx-CAM or Seprafilm) ÷ (weight of dry Cx-CAM or Seprafilm)] × 100.

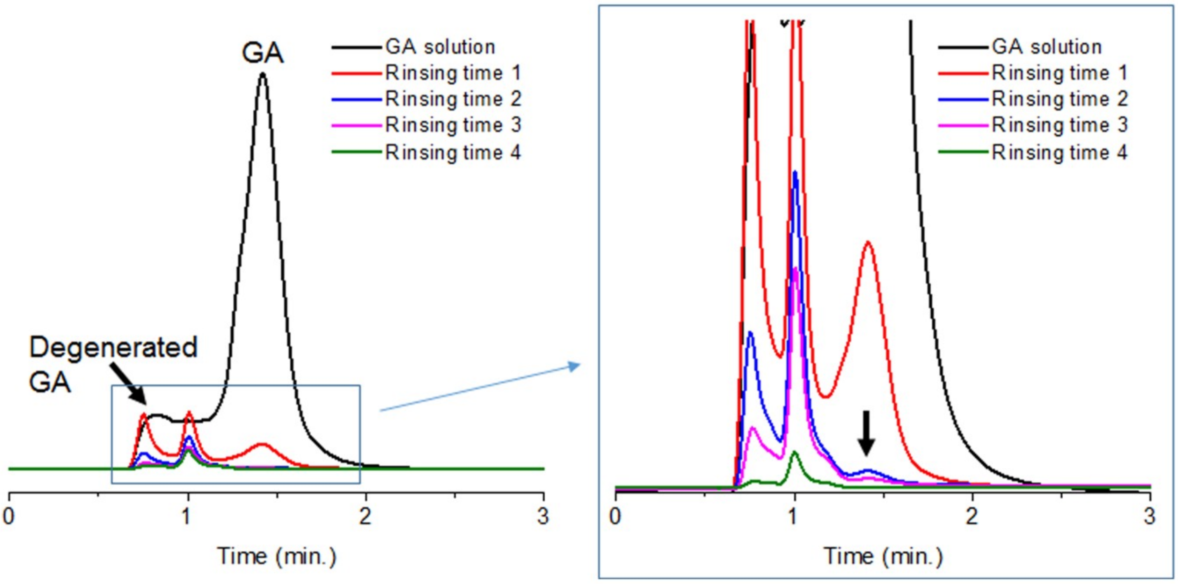

Figure S1. GA amount remained in PBS after washing of Cx-CAM films.

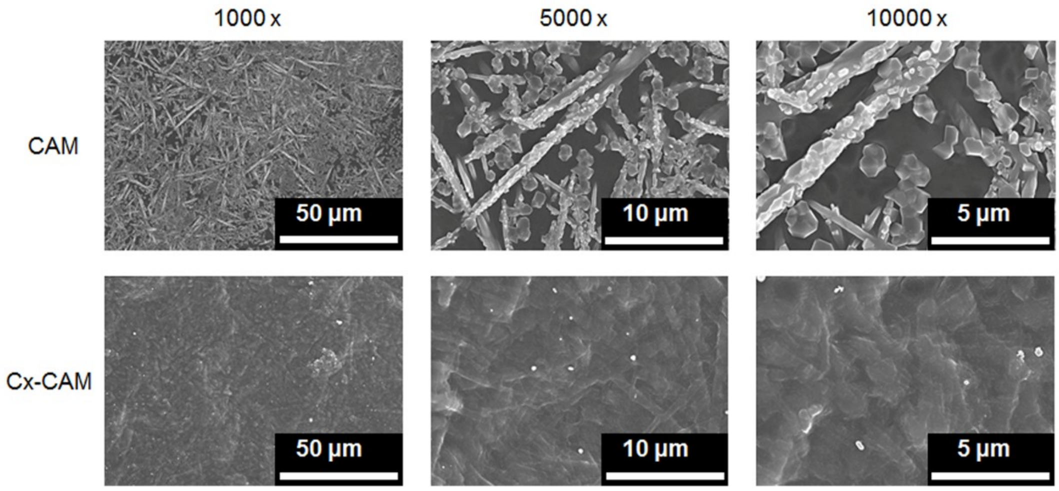

Figure S2. SEM images of CAM and Cx-CAM.

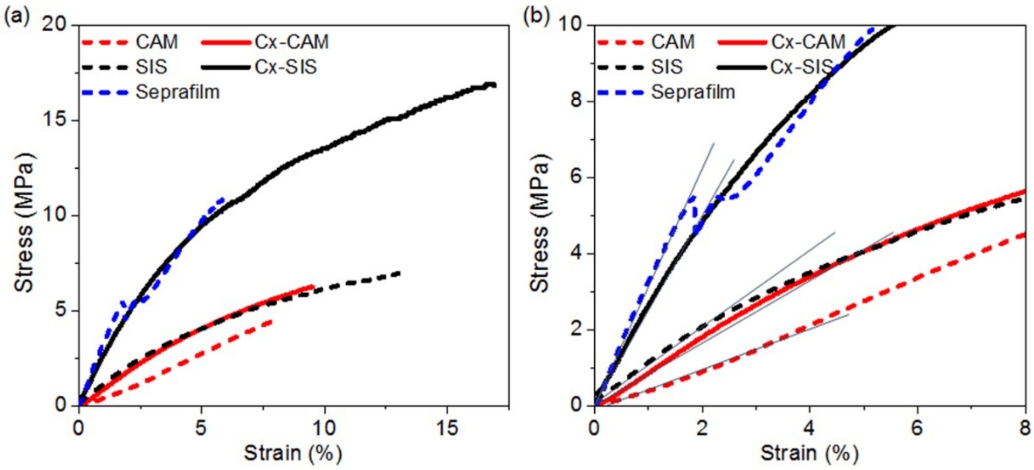

Figure S3. (a) Stress–strain curves and (b) enlarged curve of CAM, Cx-CAM, SIS, Cx-SIS films and Seprafilm.

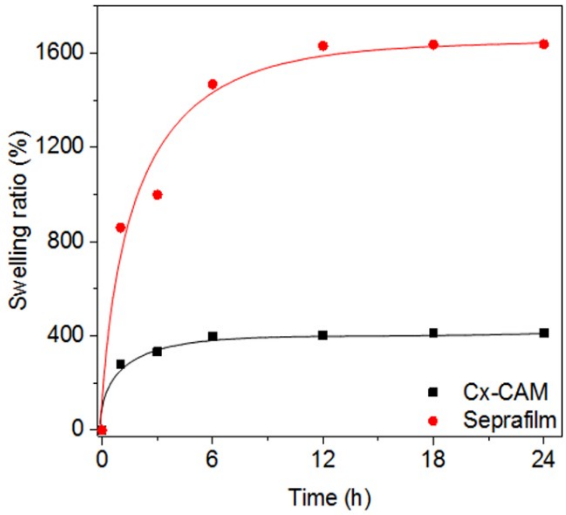

Figure S4. The swelling ratios of obtained Cx-CAM film and Seprafilm.
